# Integrative blood-derived epigenetic and transcriptomic analysis reveals the potential regulatory role of DNA methylation in ankylosing spondylitis

**DOI:** 10.1186/s13075-021-02697-3

**Published:** 2022-01-05

**Authors:** Min Xiao, Xuqi Zheng, Xiaomin Li, Xinyu Wu, Yefei Huang, Qiujing Wei, Shuangyan Cao, Jieruo Gu

**Affiliations:** Department of Rheumatology, The Third Affiliated Hospital of Sun Yat-sen University, 600 Tianhe Road, Tianhe District, Guangzhou, Guangdong Province People’s Republic of China

**Keywords:** Ankylosing spondylitis, DNA methylation, Epigenetics, mRNA expression, Transcriptome

## Abstract

**Background:**

The currently known risk loci could explain a small proportion of the heritability of ankylosing spondylitis (AS). Epigenetics might account for the missing heritability. We aimed to seek more novel AS-associated DNA methylation alterations and delineate the regulatory effect of DNA methylation and gene expression with integrated analysis of methylome and transcriptome.

**Methods:**

Epigenome-wide DNA methylation and mRNA expression were profiled in peripheral blood mononuclear cells (PBMCs) from 45 individuals (AS: health controls (HCs) = 30:15) with high-throughput array. The methylome was validated in an independent cohort (AS: HCs = 12:12). Pearson correlation analysis and causal inference tests (CIT) were conducted to determine potentially causative regulatory effects of methylation on mRNA expression.

**Results:**

A total of 4794 differentially methylated positions (DMPs) were identified associated with AS, 2526 DMPs of which were validated in an independent cohort. Both cohorts highlighted T cell receptor (TCR) signaling and Th17 differentiation pathways. Besides, AS patients manifested increased DNA methylation variability. The methylation levels of 158 DMPs were correlated with the mRNA expression levels of 112 genes, which formed interconnected network concentrated on Th17 cell differentiation and TCR signaling pathway (LCK, FYN, CD3G, TCF7, ZAP70, CXCL12, and PLCG1). We also identified several cis-acting DNA methylation and gene expression changes associated with AS risk, which might regulate the cellular mechanisms underlying AS.

**Conclusions:**

Our studies outlined the landscapes of epi-signatures of AS and several methylation-gene expression-AS regulatory axis and highlighted the Th17 cell differentiation and TCR signaling pathway, which might provide innovative molecular targets for therapeutic interventions for AS.

**Supplementary Information:**

The online version contains supplementary material available at 10.1186/s13075-021-02697-3.

## Background

Ankylosing spondylitis (AS), the archetype of spondyloarthritis (SpA), is a common, highly heritable inflammatory arthritis characterized by chronic inflammatory back pain and the involvement of the axial skeleton. It is well known that susceptibility and severity of AS are largely genetically determined. Over the past decade, genetic studies of AS have provided major insights into the etiopathogenesis of the disease. Apart from the major histocompatibility complex (MHC) I allele HLA-B*27, more than 113 non-MHC genetic associations have been identified, such as genes involved in the interleukin (IL)-23/IL-17 signaling pathway [1]. However, most of the AS susceptibility loci are undefined and located in non-coding regions [2]. Despite the numerous disease-associated variants identified, they cumulatively explain only a small proportion (< 28%) of the heritability of the disease. The “missing heritability” has been proposed to come from several mechanisms, including unknown gene–gene and gene–environmental interactions, rarer genetic variants, copy number variation, or epigenetics [3].

Epigenetics refers to functional modifications to DNA without sequence alteration, which is heritable from one cell cycle to the next, potentially reversible, and is largely responsible for the cell-specific expression of genes [4]. The modifications include histone modifications, DNA methylation, and non-coding RNAs. DNA methylation, the best studied and understood epigenetic modification, plays a critical role in the regulation of gene transcription and nuclear organization and ultimately influences cellular function. Disruption of the methylome can result in aberrant gene expression, which in turn might favor the development of the disease. Many environmental risk factors, such as diet, cigarette, and infection [5, 6], have been proved to be related to the development of AS disease. Since environmental factors influence DNA methylation profiles, it may provide an essential link between genes and the environment.

Thus far, emerging data suggest that DNA methylation plays an important role in the pathogenesis of AS. Several DNA methylation studies in AS have been carried out, and some inflammatory-related genes have been identified, including SOCS1, DNMT1, BCL11B, IRF8, and IL12B [7–11]. However, these studies concentrated on known AS-genetically associated and/or inflammatory genes and methylation-specific PCR (MSP) method was frequently used, which were underpowered due to single-gene coverage. As more high-throughput methods for analyzing DNA methylation are available, an epigenome-wide association study was conducted using high-throughput microarray technology, Illumina 450K with 5 AS patients, and 5 HCs. A total of 1915 differentially methylated positions (DMPs) were identified, and the HLA-DQB1 gene achieved the most significant signal [12]. Apart from low-throughput methods used in most studies, some limitations of the aforementioned studies hampered interpretation of the results and limited applicability of the findings, including the small size of the cohorts and absence of further validation in independent cohort. Furthermore, integration of epigenetics with other “omics” data, which could improve our understanding of AS pathogenesis, is still lack.

With the advent of large-scale, base-resolution methylation technologies, we analyzed epigenome-wide DNA methylation changes using a more high-throughput microarray technology, Infinium MethylationEPIC array, to seek more novel AS-associated DNA methylation alterations. To delineate the regulatory effect of DNA methylation and gene expression, we performed an integrated analysis of epigenome-wide methylation array and RNA expression. The findings may provide better insight into pathological mechanism for AS and facilitate the development of new targets for intervention.

## Materials and methods

### Study participants and sample preparation

Cohorts participating at the discovery stage included 45 individuals (AS: health controls (HCs) = 30:15). A total of 24 subjects (AS: HCs = 12:12) were recruited in the validation stage. All patients fulfilling the 1984 modified New York criteria for AS [13] were retrospectively enrolled from the Third Affiliated Hospital of Sun Yat-sen University between 2017 and 2020. The study was approved by the ethics committee of the Third Affiliated Hospital of Sun Yat-sen University ([2013]2-93-01) and was carried out in compliance with the Helsinki Declaration. All participants signed the written informed consent about the probable use of the blood samples prior to sample collection.

Whole peripheral blood samples were collected from all patients and controls, and then diluted with phosphate-buffered saline, laid with Lymphocyte Separation Medium (TBD Sciences), and centrifuged under 2500 rpm/min for 15 min to separate peripheral blood mononuclear cells (PBMCs). Genomic DNA was extracted from blood samples with MAGEN DNA Kit (MAGEN Inc., China). Total RNA was isolated from PBMCs by standard phenol-chloroform extraction using Trizol reagent (Invitrogen Life Technologies, Inc., USA). The quality and concentration of extracted DNA and RNA were determined by NanoDrop ND-2000 Spectrophotometer (Thermo Fisher Scientific Inc., USA), and the integrity was analyzed by agarose gel electrophoresis. The extracted DNA was stored at −80°C until DNA methylation analysis. All procedures were performed according to the manufacturer’s protocols.

### Genome-wide DNA methylation and expression profiling

Infinium MethylationEPIC BeadChip (Illumina, Inc., San Diego, CA, USA) array was used to analyze DNA methylation. This platform covers more than 850,000 methylation sites per sample to be interrogated at single-nucleotide resolution, covering 99% of reference sequence (RefSeq) genes. The samples were bisulfite-converted using EZ DNA Methylation-Gold™ Kit (Zymo Research, Irvine, CA, USA) and were hybridized in the array following the manufacturer’s instructions.

Statistical analysis of the microarray data was performed with R package ChAMP [14], Minfi [15]. Before analysis, the probes with a detection *p* value above 0.01, with a bead count <3 in at least 5% of samples per probe, and all probes located in chromosome X and Y were discarded. Besides, we also removed all single nucleotide polymorphism (SNP)-related probes and cross-reactive probes that are known to measure more than one site in the genome. The details of probes filtered out in each process were presented in Table S1. From the initial 865,918 CpGs, 127,755 were excluded during quality control, leaving 740,697 CpGs for further analysis. The methylation level for each CpG was represented as a *β* values. The *β* value was the ratio of the methylated probe intensity to the overall intensity (*β* = M/ (M + U+100), where *M* and *U* denoted the methylated and the unmethylated signal intensity, respectively), which ranged from 0 (nonmethylated) to 1 (completely methylated).

The total RNA was amplified and reverse-transcribed into cDNA with Quick Amp Labeling kit (Agilent Technologies, Palo Alto, CA, USA), and the mRNA expression was profiled with Agilent Customed LncRNA & mRNA Human Gene Expression Microarray (Agilent Technologies). Raw data was extracted by Agilent Feature Extraction (V10.7), and raw signal intensities were normalized using GeneSpring GX program (V11.5.1). The low-intensity mRNAs were filtered out. The log2 transformation was used for statistical analysis, and differential expression comparison intergroups were analyzed with R package limma [16]. A false discovery rate (FDR) <0.05 using the Benjamini and Hochberg method [17] was considered as a differential expression gene (DEG).

### Identification of differentially methylated positions (DMPs) and differentially variable positions (DVPs)

The probe intensity was normalized with beta-mixture quantile (BMIQ) normalization procedures [18]. The singular value decomposition (SVD) method was implemented for assessing the technical and biological variations in the methylation dataset [19]. Combat method [20] was used to remove the technical variations (such as batch effects). The differential methylation comparisons of AS patients versus the health donors were conducted with the limma package. A β difference (Δβ) >0.05 and an FDR <0.05 in *t* test was determined as the threshold for DMPs in our study. Among DMPs, the sites elevating in AS patients were designated as hypermethylated positions and designated as hypomethylated positions otherwise. The promoter regions were defined as TSS1500, TSS200, and 5′UTR according to the Illumina annotation file.

Owing to the evidence that the methylation alterations of disease exhibit a more heterogeneous pattern, we also detected the differentially variable and methylated CpGs (DVPs) in the current study using the recently developed iEVORA algorithm [21]. This algorithm employs Bartlett’s test (FDR < 0.001) in combination with a *t* test (*p*<0.05) to identify the DVPs. The modified test was superior to Bartlett’s test because Bartlett’s test is overly sensitive to single outliers.

### Gene ontology (GO) analysis, pathway, and transcription factor (TF) motif enrichment analysis

GO enrichment analysis and Kyoto Encyclopedia of Genes and Genomes (KEGG) biological pathways were performed using gometh function in missMethyl package [22]. To identify the enriched TF motifs for the DMPs, the HOMER method in ELMER package was used to find motif occurrences in a ±250bp region around DMPs. The DMPs which locate 2Kb away from TSS (called distal probes) were target probe set and all distal probes in the BeadChip were considered as background.

### Statistical analysis

To determine whether the methylation levels were associated with gene expression of genes which the DMP physically located within TSS1500, TSS200, 5′UTR, gene body, or 3′UTR (cis-regulation), the Pearson correlation test was conducted. Furthermore, to infer the potentially causative regulatory effects of methylation, a causal inference test (CIT) was employed [23]. This analysis calculates the likelihood of whether CpGs (causal factor) causes AS disease (outcome) via gene expression (mediator) by four statistical tests: step (1) DNA methylation is associated with AS after adjusting for age, gender; step (2) mRNA expression is associated with AS after adjusting for age, gender; step (3) DNA methylation is associated with expression after adjusting for age, gender, and AS; and step (4) DNA methylation is independent of AS after adjusting for age, gender, and expression. The multivariate linear regression was applied in each step and only simultaneously satisfied the FDR adjusted *p* values less than 0.05 in steps 1–3 and greater than 0.05 in step 4 can we conclude the potential causality. Data were analyzed with R software (Version 3.6.1, https://www.r-project.org). Statistical significance was set at a *p* value less than 0.05, except as indicated.

## Results

### PBMCs from AS patients displayed aberrant DNA methylome

**Fig. 1:**
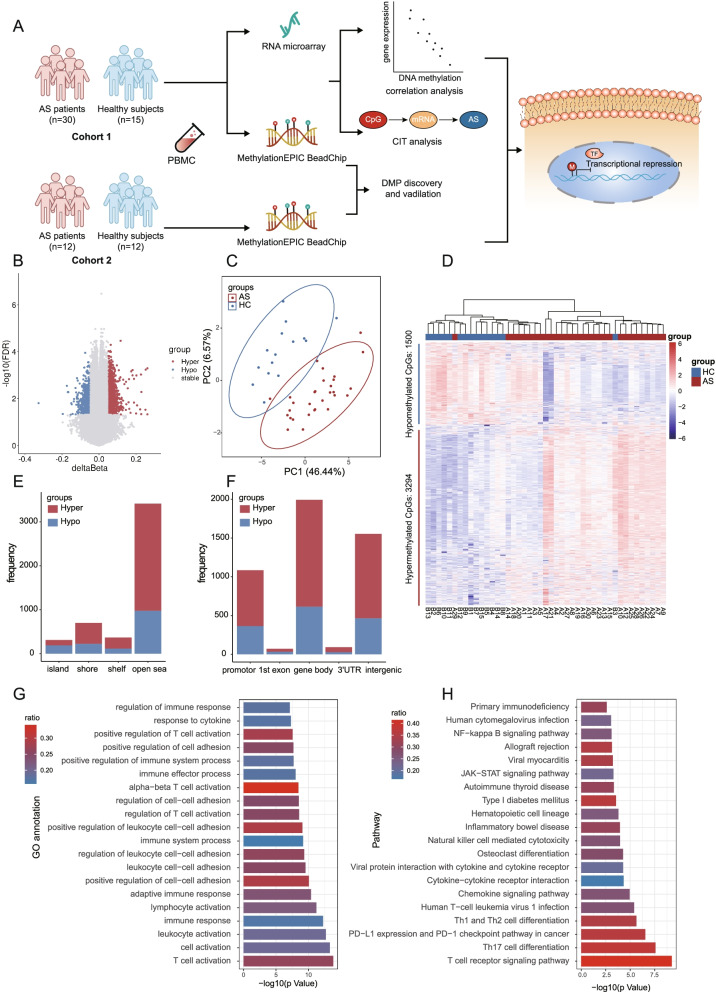
Characteristics of the differential DNA methylation positions (DMPs) associated with AS in the discovery cohort. **A** Flow diagram of the study design and analytical pipeline. **B** Volcano plot of Δβ vs. [-log_10_ (FDR)] of the filtered 740697 probes. Red dots and blue dots denote hypermethylated (*n*= 3294) and hypomethylated probes (*n*=1500) (FDR <0.05 and Δβ >0.05). **C** Principal component analysis (PCA) of DMPs show separation of AS cases form health controls (HCs). **D** DNA methylation heatmap showing DMPs between AS patients and HCs. **E** The distribution of hypermethylated and hypomethylated DMPs relative to CpG island regions. Shores are defined as the 2 kb away from CpG island and shelves as the 2 kb outside of a shore. Regions outside this 4 kb stretch are referred to as the “open sea.” **F** Genomic location of the hypermethylated and hypomethylated DMPs relative to promoters, 1st Exon, gene body, 3′UTR, and intergenic. **G** Representation of the top 20 Gene ontology (GO) biological process terms of DMPs. **H** Representation of the top 20 KEGG biological pathways associated with DMPs. Ratio represents the percentage of DMPs in each term or pathway. CIT, causal inference test; TF, transcription factor. FDR, false discovery rate

The flow diagram of the study design and analytical pipeline was depicted in Fig. 1A. Here, we described a genome-wide DNA methylation analysis and RNA microarray analysis comprising a cohort of 30 AS patients and 15 controls, in which we aim to identify AS-associated DNA methylation changes in PBMCs and further to study the effect of DMPs on the gene expression. Moreover, we also validated the DNA methylation alteration in an independent cohort. The basic demographic and clinical characteristics of cohort 1 at the time of blood sampling were presented in Additional file 1, Table S2. Overall, most AS patients and healthy volunteers were male (90% vs. 80%, *p*=0.384) and the ages were matched (34.53 ± 9.92 vs. 32.13 ± 9.42, *p*=0.441). More patients were HLA-B27 positive comparable to controls (76.7% vs. 40.0%, *p*=0.015).

Following filtering procedures, 740,697 probes were retained for further analysis. Latent confounding factors including technical variations (such as the samples position on the BeadChip), gender, age, and HLA-B27 status were included in the SVD to assess the significant components of variation (Additional file 2, Figure S1A and S1B). To reduce batch effect, we designed the similar proportion of two groups samples in two plates in the study design. However, the results suggested that overall methylome contributions to AS disease were not associated with the gender, age, or HLA-B27 status but with sample groups and position on the array (such as array plate and slide), which emphasizes the significance of sample group in DNA methylation difference in the raw data. After correcting the covariates including array plate and slide, which did not confound with the same group, only the sample group variance was captured by the top principal component (PC1, 41.5%, *p*<0.05). The PCA analysis based on top 1000 most variable CpG sites showed no apparent distinction between two groups (Additional file 2, Figure S1C), but there was a separation trend. The epigenome-wide association analysis identified 4794 DMPs, including 3294 (68.7%) hypermethylated and 1500 (31.3%) hypomethylated positions in AS patients (Fig. 1B). The complete list of significant DMPs is provided in online supplementary data S1. The identified DMPs allowed a clear distinction of most AS cases from controls in the principal component analysis (PCA) (Fig. 1C) and unsupervised hierarchical clustering (Fig. 1D). According to the genomic annotation, the AS DMPs were mapped to 2256 differentially methylated genes (DMGs). DMPs were predominantly located in open sea and gene body, only 6.5% and 22.6% DMPs were in CpG island and promoter regions (Fig. 1E, F).

GO analysis demonstrated that the DMGs were associated with several biological processes (BP) in AS, including T cell activation, immune response, and the regulation of cell-cell adhesion (Fig. 1G). KEGG pathway analysis of AS-associated epi-signatures enriched in T cell receptor (TCR) signaling pathway, Th17 differentiation, Th1 and Th2 cell differentiation, osteoclast differentiation, and JAK−STAT signaling pathway (Fig. 1H), some of which were proposed to involve in the development of AS [24]. On further inspection of the DMPs related gene in above pathways, several genes with differential methylation were confirmed to be associated with AS in previous GWAS study (Additional file 2, Figure S2), such as STAT3 (cg24312520, cg17833746, and cg05487134) [25], IL12RB2 (cg14849855, cg09018107, and cg02566391) [26], ERAP2 (cg01955050 and cg26194172), RUNX3 (cg03961551, cg25616056, cg27058497, cg13461622, and cg09993145) [27], NFKB1 (cg27333178 and cg15483813), TNF (cg24452282, cg20477259, and cg15989608), ANKH (cg14882828, cg07573020, cg08838149) [28], and LTBR (cg23079808) [29]. The results support the potential functional impact of GWAS SNPs in AS. Altogether, these observations highlight an implication of the DMPs in the pathogenesis of AS.

### AS patients manifested increased DNA methylation variability

**Fig. 2:**
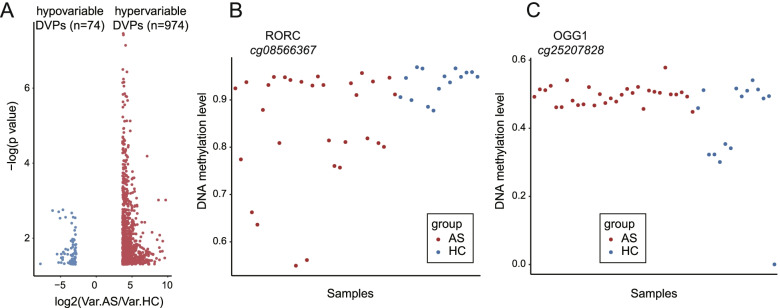
Analysis of differential variable positions (DVPs) identified with iEVORA algorithm between AS patients and HCs. **A** Volcano plot of the effect size [log_2_ (variation ratio of AS compared to HCs)] vs. [-log_10_ (*p* value)]. Red dots and blue dots denote the hypervariable probes (*n*= 974) and the hypovariable probes (*n*=74) with the *p* value of *t* test below 0.05 and FDR of Bartlett’s test below 0.001. **B**, **C** The methylation level (*β* values) of the two positions displaying methylation variability in the AS case and control samples

As previously reported, a higher heterogeneity of DNA methylation profiles was implicated in tumors and rheumatoid arthritis [21, 30]. Hence, we applied the recently developed iEVORA algorithm to test the methylation variability between AS patients and healthy subjects. A total of 1048 DVPs were identified, the majority of which (974, 92.9%) were hypervariable in AS, while only 74 DVPs were hypovariable (Fig. 2A and online supplementary data S2). The increased DNA methylation variability in disease was in line with the previous observation in other diseases, indicating the intrinsic heterogeneity in AS patients, which might be influenced by diverse factors, such as disease activity and treatment. Figure 2B gave the examples of hypervariable and hypovariable methylation positions in AS patients.

### Validation of the DNA methylome profiles in an independent cohort

To validate the DNA methylome changes in AS, we also performed the genome-wide methylation profiles in an independent cohort consisting of 12 AS patients and 12 HCs. The demographic characteristics of validation cohort were presented in Additional file 1, Table S3. After probe filtering and normalization, a clear separation was presented in PCA plot based on the top 1000 most variable probes amongst all samples, indicating the different methylated profiling between AS and HCs again. A total of 116,003 DMPs were identified with the same cutoff value of cohort 1 (FDR<0.05 and Δβ ≥ 0.05). To determine robust methylation signatures of AS, a more stringent threshold (FDR<0.05 and Δβ ≥ 0.1) was applied. As a result, 31,741 DMPs which mapped to 8531 differential methylated genes were detected, with 13,385 upregulated and 18,356 downregulated in AS. Hierarchical clustering and PCA demonstrated a greater distinction between AS patients and HCs than cohort 1 (Additional file 2, Figure S3A, S3B). The location of the DMPs in CpG island and gene position was similar to that of the discovery cohort (Additional file 2, Figure S3C, S3D). Both cohorts share 2526 DMPs and 1753 DMGs in common (*p*= 5.6×10^-09^ and 6.1×10^-14^, Additional file 2, Figure S4A, S4B). Six out of the top 20 enriched GO terms based on DMGs were overlapped, such as leukocyte activation, immune response, lymphocyte activation, immune system process, immune effector process (*p*= 2.2×10^-16^, Additional file 2, Figure S4C). Besides, 10 out of the top 20 enriched KEGG pathways were shared in two cohorts, including TCR signaling pathway, Th17 cell differentiation, chemokine signaling pathway, osteoclast differentiation (*p*=2.2×10^-16^, Additional file 2, Figure S4D). The aforementioned AS-associated DMPs were also repeated with the same direction in the validation cohort (Additional file 2, Figure S5). The independent validation confirmed the robust alteration of DNA methylation in AS patients.

### DNA methylation has regulatory effect on mRNA expression in AS patients

**Fig. 3:**
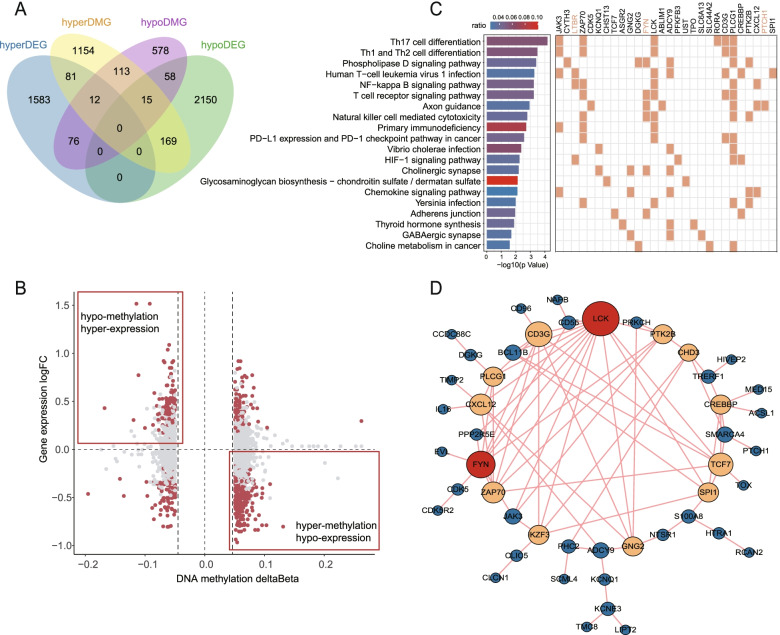
The integrating analysis of individual-level DNA methylation and mRNA expression data. **A** Venn diagram depicting differentially methylated positions related genes (DMGs) and differentially expressed genes (DEGs) between AS cases and health controls (HCs). **B** Starburst plot of gene expression changes (log2 FC) vs. DNA methylation changes (Δβ). Red dots represented the probes with significantly differential methylation and differential expression. FC, fold change. **C** Top 20 KEGG biological pathways of 112 DEGs whose expression levels are correlated with the corresponding methylation levels. The orange squares denote that gene present in the pathway. **D** The protein-protein interactions network among the correlated genes with STRING software. The sizes of the nodes are correlated with the degree (the connected number). The blue, orange, and the red denote the degree from 1 to 4, from 5 to 9, above 10, respectively

To better understand the regulatory effect of methylation in AS, we integrated the individual-level DNA methylation and mRNA expression data. The expression data of 31 AS patients and 20 age and gender-matched HCs were detected with customed Gene Expression Microarray. With the threshold of FDR <0.05, a total of 4144 DEGs were identified, of which 1752 genes were highly expressed and 2392 genes were lowly expressed in AS patients (online supplementary data S3). First, we examined the intersection of differentially methylated and differentially expressed genes. As shown in the Venn plot (*p*=1.1×10^-07^, Fig. 3A), a total of 411 genes covering 623 CpGs manifested differential DNA methylation and RNA expression simultaneously. The integrating analysis demonstrated that the inverse association was more common between methylation and expression, and most genes were hyper-methylated and hypo-expressed (Fig. 3B), which supported the recognized “switch” role of DNA methylation for gene silencing.

To define the precise regulatory association of methylation on mRNA expression, the pairwise Pearson correlation analysis were performed between the overlap 628 DMPs and proximity 411 DEGs, namely the DMPs located in the positional window including TSS1500, TSS200, 5′UTR, gene body, and 3′UTR of the DEGs, among the sample set who had detected DNA methylation as well as mRNA expression (AS=26, HCs=9). As a result, the methylation levels of 158 DMPs were correlated with the corresponding mRNA expression levels of 112 genes (*p*<0.05) (cis-regulation) (online supplementary data S4). Negative correlation dominated the 158 cis-regulated methylation-mRNA pairs (negative: positive= 127:31), which was also consistent with the gene silencing role of DNA methylation.

Pathways of correlated DMPs were enriched in Th17 cell differentiation, Th1 and Th2 cell differentiation, phospholipase D signaling pathway, NF-kappa B signaling pathway, and TCR signaling pathway (Fig. 3C). Of note, several genes have widely involved these disturbed pathways, such as LCK, FYN, ZAP70, CD3G, JAK3, LTBR, and PLCG1. Besides, we constructed the protein-protein interactions (PPI) network among the 112 correlated genes with STRING software (Fig. 3D). The most interacted hub genes were LCK, FYN, CD3G, TCF7, ZAP70, CXCL12, and PLCG1.

**Fig. 4:**
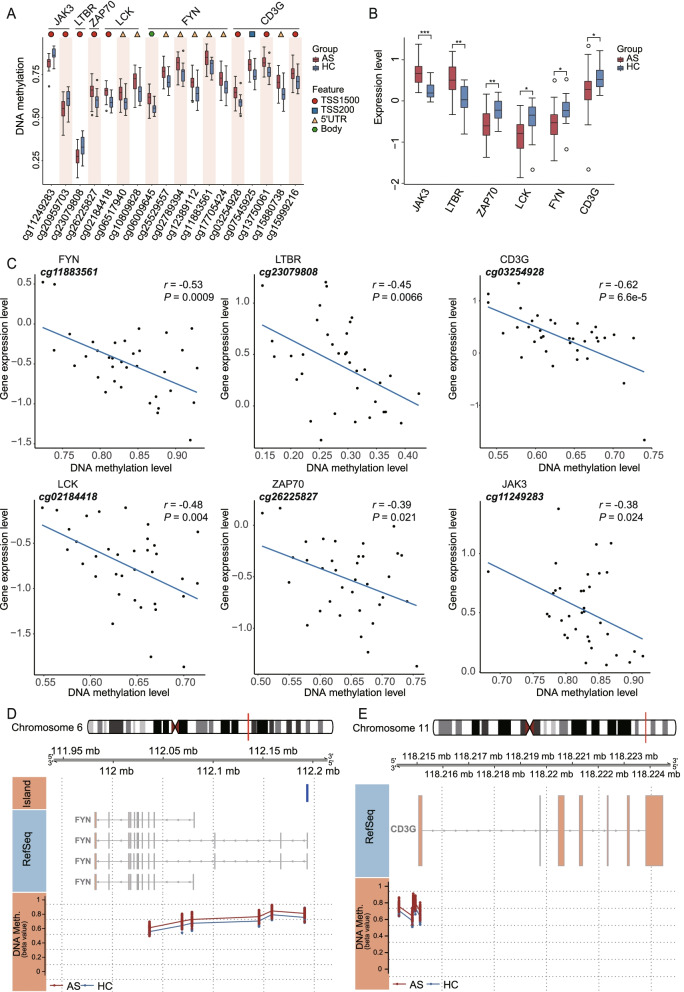
DNA methylation and RNA expression profiles of the identified hub genes in the integrating analysis. **A** Box plots showing the methylation level of CpGs in 6 differentially expression genes. The locations of the CpGs are shown in at the top of the boxplots. **B** Box plots showing the expression level of 6 differentially expression genes. For box plots, center lines represent the median, box edges represent the 25th and 75th percentiles, whisker represents the percentile ± 1.5 times interquartile range, and the dots represent the outliers. Difference between the two groups was tested by Mann–Whitney *U* test. *P < 0.05; **P < 0.01; ***P < 0.001. **C** The correlation scatter plots of expression levels against methylation levels for 6 differentially expression genes in AS cases. r, Pearson correlation coefficient. **D**, **E** The overview of genomic organization and differential methylation sites of FYN (**D**) and CD3G (**E**). The first track depicts the chromosome, with the gene region highlighted in red box. The second track represents the genome coordinate. The third track represents the transcripts of the gene. The bottom track shows the methylation level of the CpGs along the gene between AS cases and controls

The DMPs related to the key genes in the discovery cohort were shown in Fig. 4A, which were verified in the validation cohort (online supplementary data S5). The expression levels of two Src family tyrosine kinases, FYN and LCK, were decreased in AS patients, and inversely, the methylation levels of 6 CpGs in FYN and 3 CpGs in LCK, most of which located in promotor regions, were elevated in AS patients (Fig. 4A, B). CD3G and ZAP70 were with decreased expression and increased methylation level of 5 and 1 promotor-located CpGs. JAK3 and LTBR had 2 and 1 decreased DMPs located in TSS1500, respectively, and the expression levels were significantly upregulated in AS patients (Fig. 4A, B). Figure 4C demonstrated a significantly negative correlation between the methylation and expression of these genes. The overview of genomic organization and methylation sites of FYN and CD3G was illustrated in Fig. 4D, E, the differential sites clustered surrounding the promotor region. Collectively, these observations indicated that the DNA methylation had a regulatory effect on the mRNA expression of predisposing genes in AS.

### Causal inference tests and transcription factor motif enrichment analysis

The correlation analysis could not determine the direction of causality of the strong association between methylome and transcriptome; therefore, we conducted the CIT analysis to infer the cis-acting DNA methylation and gene expression changes associated with AS risk. Among the 158 correlated DMP-DEG pairs, we identified 17 negatively correlated pairs corresponding 12 genes satisfying the four steps (Table 1). Notably, FYN, which widely interacted with other genes in the PPI network and involved in several significant pathways, was also highlighted with the differential expression potentially regulated by five DMPs (cg02789394, cg06009645, cg11883561, cg12389112, cg25529557). Besides, the aberrant expression of LCK in PBMC was mediated by promoter hypomethylation (cg02184418). Moreover, the CIT analysis also suggested the potentially causal role of other 11 CpGs.

**Table 1:** The gene identified in CIT analysis for which transcriptomics likely mediates ankylosing spondylitis (AS) risk of DNA methylation

Gene	DMPs			DEGs		Correlation		CIT			
	CpGs	△β	FDR	FC	FDR	r	p	p1	p2	p3	p4
FYN	cg02789394	0.056	0.012	0.80	0.019	−0.51	0.002	0.026	0.019	0.040	0.147
	cg06009645	0.054	0.007			−0.45	0.006	0.022	0.025	0.040	0.132
	cg11883561	0.053	0.019			−0.53	0.001	0.049	0.015	0.040	0.288
	cg12389112	0.060	0.013			−0.50	0.002	0.043	0.019	0.040	0.227
	cg25529557	0.061	0.007			−0.47	0.004	0.026	0.025	0.040	0.141
LCK	cg02184418	0.058	0.002	0.76	0.012	−0.480	0.004	0.011	0.019	0.036	0.097
TBC1D10C	cg17161520	0.059	0.016	0.66	0.003	−0.530	0.001	0.038	0.015	0.019	0.339
TCF7	cg20682563	0.053	0.005	0.71	0.008	−0.460	0.005	0.014	0.022	0.047	0.097
CCDC88C	cg07112604	0.053	0.001	0.78	0.007	−0.514	0.002	0.005	0.019	0.030	0.062
	cg13873287	0.060	0.007			−0.463	0.005	0.023	0.019	0.030	0.147
EVL	cg20407362	0.057	0.012	0.68	0.000	−0.567	0.000	0.030	0.011	0.010	0.398
NUP210	cg06143615	0.053	0.003	0.84	0.009	−0.528	0.001	0.011	0.015	0.036	0.097
HTRA1	cg06474225	−0.060	0.030	2.12	0.001	−0.570	0.000	0.032	0.010	0.025	0.334
PMEPA1	cg07538640	0.059	0.010	0.69	0.012	−0.490	0.003	0.026	0.019	0.030	0.176
PRKCH	cg22061465	0.062	0.003	0.68	0.010	−0.506	0.002	0.013	0.019	0.026	0.130
PTCH1	cg20445630	0.057	0.006	0.75	0.022	−0.514	0.002	0.032	0.016	0.020	0.271
RFPL2	cg01124132	0.096	0.018	0.73	0.013	−0.553	0.001	0.032	0.011	0.015	0.345

*DMP* differentially methylated positions, *DEG* differentially expressed gene, *CIT* causal inference test, *Δβ* difference of methylation between patients with AS and healthy controls, *FDR* false discovery rate, *FC* fold change, *r* Pearson correlation coefficient, *p1-4* the significance of the test in step 1-4 in CIT analysis, respectively

**Fig. 5:**
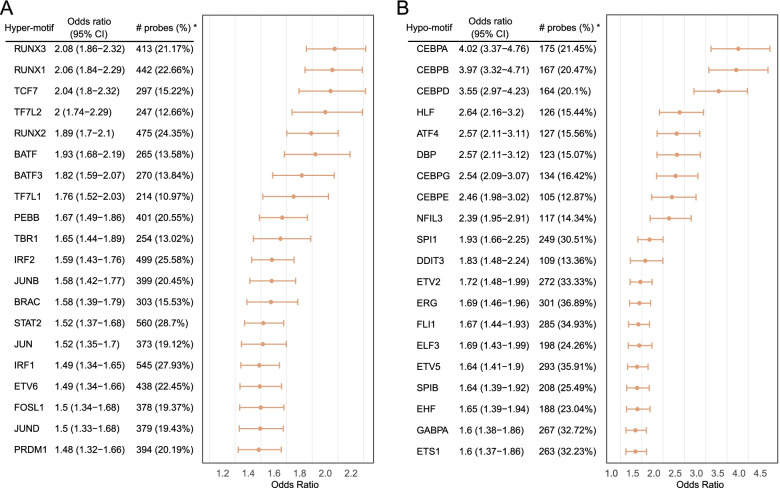
Forest plots showing enrichment analysis of transcription factor (TF) motif with HOMER method. **A** and **B** denoted the hypermethylated and hypomethylated CpGs set. The top 20 enriched TF motif are shown. TF enrichments were quantified using Fisher’s exact test. *The percentage of the probes containing a motif occurrence in hypermethylated or hypomethylated distal-probes. The error bars represent 95% confidence interval of odds ratio

DNA methylation can be used to identify functional changes at transcriptional enhancers. Therefore, we performed the TF motif enrichment analysis with the DMPs falling into distal feature regions (distal-probes). As a result, a total of 1951 hypermethylated and 816 hypomethylated distal-probes were identified. The enrichment analysis revealed that 57 TF motifs and 40 TF motifs were significantly enriched in the hypermethylation and hypomethylation set, respectively. The top 20 motifs were presented in Fig. 5. For the hypermethylation set, Runt-related factors, TCF-7-related factors, and interferon-regulatory factors were significantly enriched. For the hypomethylation set, C/EBP family together with ETS-related factors family were enriched. We also observed the expression difference of TF in the C/EBP family, including CEBPB, CEBPD, and NFIL3. The expression of C/EBP TF family members could be modulated by several cytokines through several downstream pathways, such TNF, IFNs, and IL 17, which had been recognized to be involved in AS [31].

## Discussion

The susceptibility to AS has a strong genetic component dominated by MHC allele HLA-B*27. The polygenic nature of AS has been slowly unraveled over time with more than 100 loci identified. However, a small proportion of the heritability could be explained by currently known risk loci. Several mechanisms, such as epigenetics, have been proposed to account for the “missing heritability.” Thus, our study carried out a genome-wide DNA methylation analysis, along with integration with transcriptomic analysis. In this study, we demonstrated the alteration of DNA methylome and gene transcriptome in PBMCs underlying predisposition to AS. We also identified several cis-acting DNA methylation and gene expression changes associated with AS risk, which might regulate the cellular mechanisms underlying AS. Moreover, we described the perturbated methylation in the highly interconnected network concentrated on Th17 cell differentiation and TCR signaling pathway.

Methylation of promoter CpGs favors a closed chromatin conformation and blocks transcription factors, resulting in negatively regulatory effect on mRNA expression. Correlation analysis in our study demonstrated that negative correlation dominated the methylation-mRNA pairs, which favored the “silencing” epigenetic mark of DNA methylation. The integrating analysis identified several genes with aberrant and correlated methylation and expression level, including LCK, FYN, CD3G, ZAP70, PLCG1, LTBR, JAK3, TCF7, and CXCL12, which particularly concentrated on Th17 cell differentiation, Th1 and Th2 cell differentiation, and TCR signaling pathway. Apart from integrating analysis, TCR signaling pathway was also highlighted in pathway enrichment analysis of all DMPs in two cohorts. LCK and FYN are the predominant Src family kinases found in T lymphocytes, the activation of which is central to the initiation of TCR signaling pathways. Active LCK or FYN can phosphorylate the CD3, resulting in the recruitment of the ζ-chain-associated protein of 70 kDa (ZAP70) kinase, further recruits and facilitates the activation of further downstream kinases. TCR proximal signaling via the Src-family kinases, LCK and FYN, can influence T cell activation, differentiation, and tolerance [32]. The CIT analysis showed that several DMPs of FYN had regulatory effects on mRNA expression and consequently increased disease susceptibility. FYN has been reported to promote osteoarthritis by activating the β-catenin pathway [33]; nevertheless, its role in the development of AS has yet to be determined. To our knowledge, imbalance of immune cells, including Th1, Th2, Th17, and Treg cells, was relevant to AS [34]. Curdlan-treated SKG mice which possess ZAP70 mutation is a good vivo animal for AS. Abnormal ZAP70 could impaired TCR signal transduction in SKG mice and transgenic expression of the normal human ZAP70 gene in SKG mice could rescue the SKG arthritis, which emphasizes the critical function of ZAP70 [35]. Here, our study also revealed that the downregulated expression of ZAP70 and downstream molecules (such as PLCG1) are associated with AS. Therefore, we speculated these genes might contribute to the immunity imbalance in AS through insufficiently activated downstream TCR signaling pathways, and DNA methylation might involve in this regulatory mechanism besides gene mutation.

Over the past 10 years, the predisposing role of IL-17 in AS is increasingly recognized. Most strikingly, we also identified many DMGs associated with IL-23/17 axis. When IL-23 binds to its receptor complex (IL-12Rβ1/IL-23R), it subsequently recruits JAK2 and Tyk (Janus family of tyrosine kinases) and mediates STAT3 phosphorylation and facilitates differentiation into IL-17–expressing cells [36]. Th17 cells are the most common class of IL-17-producing adaptive lymphocytes [24]. Once secreted, IL-17 stimulate stromal cells and immune cells to promote inflammation through the activity of TFs such as NF-ĸβ and AP-1 [37]. Besides, IL-17 is known to alter the activity of osteoclasts and osteoblasts, probably contributing to aberrant bone formation [24]. Several genes in IL-23/IL-17 axis were reported association with AS in GWAS studies, including IL23A, IL23R, IL12RB2 [26], STAT3 [25], and NFKB1 [38], the CpG sites of which were found dysregulated in our study. Furthermore, several gene, with correlated expression and methylation level, involved in Th17 differentiation (Fig. 3C), including JAK3, ZAP70, LCK, RORA, CD3G, and PLCG1, most of which were also hub genes in PPI network (Fig. 3D). Given widely perturbations of DNA methylation in IL-23/IL-17 axis in AS and motivated by success of IL-17 inhibitor in AS, other therapeutic targets in this axis deserve further test.

LTBR (lymphotoxin beta receptor), a member of the TNF receptor superfamily, the elevated expression and the downregulated promotor methylation were observed in AS. The previous study found that a variant in LTBR was convincingly associated with AS regardless of the evidence of involvement of TNF pathways in AS pathogenesis [29]. It was first reported the gene displayed an altered methylation in AS. ERAP2 (endoplasmic reticulum aminopeptidase 2), with two upregulated DMPs found in our study, has reported to be associated with AS [39]. ERAP2 has crucial role in trimming peptides to an optimal length for binding to class I MHC molecules [40]. The altered ERAP1 or ERAP2 activities could lead to abnormal peptide complexes, ultimately trigged the unfolded protein response (UPR) and autophagy, which is the putative mechanism in AS [24]. RUNX3 (runt-related transcription factor 3), which was strongly associated with AS by GWAS [29], were most enriched transcription factor in the hypermethylation set (Fig. 5A). Five sites of RUNX3 were hypermethylated in AS (Additional file 2, Figure S2 and S5) and 3 of which located in promoter region. The expression of RUNX3 was decreased in AS without significance (FDR=0.08) in our study; however, the previous study reported a lower expression in AS than healthy controls (*P*<0.002) [41]. We speculated that the relatively small sample size might conceal the expression difference. RUNX3 plays key roles in the development and differentiation of immune cells and two distinct SNPs appear to exert their influence in different cell types—rs4648889 in CD8+ T cells and rs4265380 in monocytes [41]. Our study indicated that the aberrant methylation might play roles in AS predisposition. Although high-throughput techniques considerably facilitate our understanding of AS pathogenesis, more progress towards the precise mechanistic explanation in cellular or animal experiments are required in future studies.

Previous studies detected several differentially methylated genes associated with AS using single-gene methylation-specific PCR, some of which have been repeated in our study. We have found 6 methylation sites in BCL11B were upregulated; moreover, the expression of BCL11B was decreased in AS patients, which was in line with the report [8]. Besides, we also detected the hypermethylation in IRF8 and SOCS1 coincident with the previous findings [9, 11]. Compared to the previous genome-wide DNA methylation profiles in AS with Illumina Infinium HumanMethylation450 BeadChip [12], we increased the sample size and adopted larger coverage arrays. Besides, we also performed validation in an independent cohort to identified the robust DNA methylome changes associated with AS. Integrating with mRNA expression data could provide better understand to the regulatory effect of methylation in AS.

Our study has several limitations. Methylation modification can be affected by demographic and lifestyle factors. In our study, the overall AS related methylome were not associated with the age, gender, and HLA-B27 status in the SVD analysis. However, the contribution of these factors could not conclude easily due to the limited sample sizes of our study and need more validation. Furthermore, the lifestyle factors (such as smoking, diet, and medication) may confound the DMP detection without correction the variates. Another limitation for our study is that we do not analysis the cell type composition of the PBMC samples as well as the methylation profiles of specific cell type, so we could not conclude whether the methylation and transcription alteration is a cause or a consequence of the cellular composition. To note, it is impossible to conclusively prove causal relationships based on observational data alone, so the aims of our study are to identify the potential regulatory axis, which proves the foundation for our further mechanistic research. Although plenty of DMGs have been confirmed to involve in the development of AS, we did not investigate whether the methylation mediated the risk of AS-associated SNPs at these loci. Integration with GWAS data should be conducted to define the methylation-mediated the regulatory mechanisms of noncoding risk variants in the future.

## Conclusions

In summary, the research of DNA methylation in AS was still in its infancy, our studies outlined the landscapes of epi-signatures of PBMC from AS patients. With integration with transcriptomic analysis, several methylation-gene expression-AS regulatory axis enhanced our comprehension of the regulatory effect of DNA methylation on mRNA expression. Our results highlight the perturbated methylation in Th17 cell differentiation and TCR signaling pathway, which might provide innovative molecular targets for therapeutic interventions for AS.

## Supplementary Information

**Additional file 1: Table S1.** The number of probes removed in six filtering programs. **Table S2.** The demographic characteristics of the participants in the discovery cohort. **Table S3.** The demographic characteristics of the participants in the validation cohort.

**Additional file 2: Figure S1.** The singular value decomposition (SVD) plots showing the correlation between covariates and primary components (PC). **Figure S2.** Box plots showing the methylation level of differentially methylated CpGs (DMPs) in the discovery cohort. **Figure S3.** Characteristics of the differential DNA methylation positions (DMPs) associated with AS in the validation cohort. **Figure S4.** Venn diagram depicting the overlaps between the discovery and validation cohorts. **Figure S5.** Box plots showing the methylation level of differentially methylated CpGs (DMPs) in the validation cohort.

**Additional file 3: Supplementary Data S1.** 4794 differentially methylated positions (DMPs) identified associated with ankylosing spondylitis in the discovery cohort. **Supplementary Data S2.** 1,048 differentially variable positions (DVPs) identified with iEVORA algorithm in the discovery cohort. **Supplementary Data S3.** 4,144 differentially expressed genes (DEGs) identified associated with ankylosing spondylitis in the discovery cohort. **Supplementary Data S4.** 158 differentially methylated positions (DMPs) identified to be correlated with the corresponding mRNA expression levels of 112 genes in Pearson correlation analyses. **Supplementary Data S5.** The differentially methylated positions (DMPs) of 6 key genes identified in the Pearson correlation analyses were verified in the validation cohort.

## Data Availability

The datasets presented in this study can be found in online repositories. The names of the repository/repositories and accession number(s) can be found below: https://www.ncbi.nlm.nih.gov/geo/, GSE179571.

## Abbreviations

AS: Ankylosing spondylitis
PBMCs: Peripheral blood mononuclear cells
HCs: Health controls
CIT: Causal inference tests
DMPs: Differentially methylated positions
TCR: T cell receptor
SpA: Spondyloarthritis
MHC: Major histocompatibility complex
IL: Interleukin
MSP: Methylation-specific PCR
RefSeq: Reference sequence
SNP: Single-nucleotide polymorphism
FDR: False discovery rate
DEG: Differential expression gene
DVPs: Differentially variable positions
BMIQ: Beta-mixture quantile
SVD: Singular value decomposition
GO: Gene ontology
TF: Transcription factor
KEGG: Kyoto Encyclopedia of Genes and Genomes
PCA: Principal component analysis
DMGs: Differentially methylated genes
BP: Biological processes
PPI: Protein-protein interactions
ASDAS: Ankylosing Spondylitis Disease Activity Score
ZAP70: ζ-chain-associated protein of 70 kDa
LTBR: Lymphotoxin beta receptor
ERAP2: Endoplasmic reticulum aminopeptidase 2
RUNX3: Runt-related transcription factor 3

## References

[1] Hanson A, Brown MA (2017). Genetics and the causes of ankylosing spondylitis. Rheum Dis Clin N Am.

[2] Wordsworth BP, Cohen CJ, Davidson C, Vecellio M. Perspectives on the genetic associations of ankylosing spondylitis. Front Immunol. 2021;12. Article number 603726. 10.3389/fimmu.2021.603726 PMC7977288 33746951

[3] Whyte JM, Ellis JJ, Brown MA, Kenna TJ (2019). Best practices in DNA methylation: lessons from inflammatory bowel disease, psoriasis and ankylosing spondylitis. Arthritis Res Ther.

[4] Costenbader KH, Gay S, Alarcón-Riquelme ME, Iaccarino L, Doria A (2012). Genes, epigenetic regulation and environmental factors: which is the most relevant in developing autoimmune diseases?. Autoimmun Rev.

[5] Lindstrom U, Exarchou S, Lie E, Dehlin M, Forsblad-d'Elia H, Askling J, Jacobsson L (2016). Childhood hospitalisation with infections and later development of ankylosing spondylitis: a national case-control study. Arthritis Res Ther.

[6] Videm V, Cortes A, Thomas R, Brown MA (2014). Current smoking is associated with incident ankylosing spondylitis -- the HUNT population-based Norwegian health study. J Rheumatol.

[7] Zhang X, Lu J, Pan Z, Ma Y, Liu R, Yang S, Yang S, Dong J, Shi X, Xu S (2019). DNA methylation and transcriptome signature of the IL12B gene in ankylosing spondylitis. Int Immunopharmacol.

[8] Karami J, Mahmoudi M, Amirzargar A, Gharshasbi M, Jamshidi A, Aslani S, Nicknam MH (2017). Promoter hypermethylation of BCL11B gene correlates with downregulation of gene transcription in ankylosing spondylitis patients. Genes Immun.

[9] Chen M, Wu M, Hu X, Yang J, Han R, Ma Y, Zhang X, Yuan Y, Liu R, Jiang G (2019). Ankylosing spondylitis is associated with aberrant DNA methylation of IFN regulatory factor 8 gene promoter region. Clin Rheumatol.

[10] Aslani S, Mahmoudi M, Garshasbi M, Jamshidi AR, Karami J, Nicknam MH (2016). Evaluation of DNMT1 gene expression profile and methylation of its promoter region in patients with ankylosing spondylitis. Clin Rheumatol.

[11] Lai NS, Chou JL, Chen GC, Liu SQ, Lu MC, Chan MW (2014). Association between cytokines and methylation of SOCS-1 in serum of patients with ankylosing spondylitis. Mol Biol Rep.

[12] Hao J, Liu Y, Xu J, Wang W, Wen Y, He A, Fan Q, Guo X, Zhang F (2017). Genome-wide DNA methylation profile analysis identifies differentially methylated loci associated with ankylosis spondylitis. Arthritis Res Ther.

[13] van der Linden S, Valkenburg HA, Cats A (1984). Evaluation of diagnostic criteria for ankylosing spondylitis. A proposal for modification of the New York criteria. Arthritis Rheum.

[14] Tian Y, Morris TJ, Webster AP, Yang Z, Beck S, Feber A, Teschendorff AE (2017). ChAMP: updated methylation analysis pipeline for Illumina BeadChips. Bioinformatics.

[15] Aryee MJ, Jaffe AE, Corrada-Bravo H, Ladd-Acosta C, Feinberg AP, Hansen KD, Irizarry RA (2014). Minfi: a flexible and comprehensive bioconductor package for the analysis of Infinium DNA methylation microarrays. Bioinformatics.

[16] Edgar R, Domrachev M, Lash AE (2002). Gene Expression Omnibus: NCBI gene expression and hybridization array data repository. Nucleic Acids Res.

[17] Benjamini Y, Hochberg Y (1995). Controlling the false discovery rate: a practical and powerful approach to multiple testing. J R Stat Soc Ser B Methodol.

[18] Teschendorff AE, Marabita F, Lechner M, Bartlett T, Tegner J, Gomez-Cabrero D, Beck S (2013). A beta-mixture quantile normalization method for correcting probe design bias in Illumina Infinium 450 k DNA methylation data. Bioinformatics.

[19] Teschendorff AE, Menon U, Gentry-Maharaj A, Ramus SJ, Gayther SA, Apostolidou S, Jones A, Lechner M, Beck S, Jacobs IJ (2009). An epigenetic signature in peripheral blood predicts active ovarian cancer. PLoS One.

[20] Johnson WE, Li C, Rabinovic A (2007). Adjusting batch effects in microarray expression data using empirical Bayes methods. Biostatistics.

[21] Teschendorff AE, Gao Y, Jones A, Ruebner M, Beckmann MW, Wachter DL, Fasching PA, Widschwendter M (2016). DNA methylation outliers in normal breast tissue identify field defects that are enriched in cancer. Nat Commun.

[22] Phipson B, Maksimovic J, Oshlack A (2016). missMethyl: an R package for analyzing data from Illumina's HumanMethylation450 platform. Bioinformatics.

[23] Millstein J, Zhang B, Zhu J, Schadt EE (2009). Disentangling molecular relationships with a causal inference test. BMC Genet.

[24] Ranganathan V, Gracey E, Brown MA, Inman RD, Haroon N (2017). Pathogenesis of ankylosing spondylitis—recent advances and future directions. Nat Rev Rheumatol.

[25] Davidson SI, Liu Y, Danoy PA, Wu X, Thomas GP, Jiang L, Sun L, Wang N, Han J, Han H (2011). Association of STAT3 and TNFRSF1A with ankylosing spondylitis in Han Chinese. Ann Rheum Dis.

[26] Roberts AR, Vecellio M, Chen L, Ridley A, Cortes A, Knight JC, Bowness P, Cohen CJ, Wordsworth BP (2016). An ankylosing spondylitis-associated genetic variant in the IL23R-IL12RB2 intergenic region modulates enhancer activity and is associated with increased Th1-cell differentiation. Ann Rheum Dis.

[27] Su W, Du L, Liu S, Deng J, Cao Q, Yuan G, Kijlstra A, Yang P (2018). ERAP1/ERAP2 and RUNX3 polymorphisms are not associated with ankylosing spondylitis susceptibility in Chinese Han. Clin Exp Immunol.

[28] Tsui HW, Inman RD, Paterson AD, Reveille JD, Tsui FWL (2005). ANKH variants associated with ankylosing spondylitis: gender differences. Arthritis Res Ther.

[29] Evans DM, Spencer CC, Pointon JJ, Su Z, Harvey D, Kochan G, Oppermann U, Dilthey A, Pirinen M, Stone MA (2011). Interaction between ERAP1 and HLA-B27 in ankylosing spondylitis implicates peptide handling in the mechanism for HLA-B27 in disease susceptibility. Nat Genet.

[30] Rodríguez-Ubreva J, de la Calle-Fabregat C, Li T, Ciudad L, Ballestar ML, Català-Moll F, Morante-Palacios O, Garcia-Gomez A, Celis R, Humby F (2019). Inflammatory cytokines shape a changing DNA methylome in monocytes mirroring disease activity in rheumatoid arthritis. Ann Rheum Dis.

[31] Schwartz DM, Bonelli M, Gadina M, O'Shea JJ (2016). Type I/II cytokines, JAKs, and new strategies for treating autoimmune diseases. Nat Rev Rheumatol.

[32] Salmond RJ, Filby A, Qureshi I, Caserta S, Zamoyska R (2009). T-cell receptor proximal signaling via the Src-family kinases, Lck and Fyn, influences T-cell activation, differentiation, and tolerance. Immunol Rev.

[33] Li K, Zhang Y, Zhang Y, Jiang W, Shen J, Xu S, et al. Tyrosine kinase Fyn promotes osteoarthritis by activating the β-catenin pathway. Ann Rheum Dis. 2018;77(6):935–43. 10.1136/annrheumdis-2017-212658 29555825

[34] Yang M, Lv Q, Wei Q, Jiang Y, Qi J, Xiao M, et al. TNF-α inhibitor therapy can improve the immune imbalance of CD4+ T cells and negative regulatory cells but not CD8+ T cells in ankylosing spondylitis. Arthritis Res Ther. 2020;22. Article number 149. 10.1186/s13075-020-02226-8 PMC7304211 32560733

[35] Sakaguchi N, Takahashi T, Hata H, Nomura T, Tagami T, Yamazaki S, Sakihama T, Matsutani T, Negishi I, Nakatsuru S (2003). Altered thymic T-cell selection due to a mutation of the ZAP-70 gene causes autoimmune arthritis in mice. Nature.

[36] Parham C, Chirica M, Timans J, Vaisberg E, Travis M, Cheung J, Pflanz S, Zhang R, Singh KP, Vega F (2002). A receptor for the heterodimeric cytokine IL-23 is composed of IL-12Rbeta1 and a novel cytokine receptor subunit, IL-23R. J Immunol.

[37] Bartlett HS, Million RP (2015). Targeting the IL-17-T(H)17 pathway. Nat Rev Drug Discov.

[38] Sode J, Bank S, Vogel U, Andersen PS, Sørensen SB, Bojesen AB, Andersen MR, Brandslund I, Dessau RB, Hoffmann HJ (2018). Genetically determined high activities of the TNF-alpha, IL23/IL17, and NFkB pathways were associated with increased risk of ankylosing spondylitis. BMC Med Genet.

[39] Reveille JD, Sims AM, Danoy P, Evans DM, Leo P, Pointon JJ, Jin R, Zhou X, Bradbury LA, Appleton LH (2010). Genome-wide association study of ankylosing spondylitis identifies non-MHC susceptibility loci. Nat Genet.

[40] Martín-Esteban A, Guasp P, Barnea E, Admon A, López DCJ (2016). Functional interaction of the ankylosing spondylitis-associated endoplasmic reticulum aminopeptidase 2 with the HLA-B*27 peptidome in human cells. Arthritis Rheum.

[41] Vecellio M, Cortes A, Roberts AR, Ellis J, Cohen CJ, Knight JC, Brown MA, Bowness P, Wordsworth BP (2018). Evidence for a second ankylosing spondylitis-associated RUNX3 regulatory polymorphism. RMD Open.

