# Supplementary material for: Integrative blood-derived epigenetic and transcriptomic analysis reveals the potential regulatory role of DNA methylation in ankylosing spondylitis
Source: Arthritis Res Ther. 2022 Jan 5;24:15. doi: 10.1186/s13075-021-02697-3 (PMC8728943; doi:10.1186/s13075-021-02697-3)
Supplement: Supplementary file 1 — Additional file 1: Table S1. The number of probes removed in six filtering programs. Table S2. The demographic characteristics of the participants in the discovery cohort. Table S3. The demographic characteristics of the participants in the validation cohort. [file 13075_2021_2697_MOESM1_ESM.docx]

Table S1. The number of probes removed in six filtering programs.

| Programs | Number of filtered probes |
| --- | --- |
| Filtering probes with a detection p-value above 0.01 | 3,327 |
| Filtering probes with a beadcount <3 in at least 5% of samples | 5,497 |
| Filtering non-CpG probes | 2,974 |
| Filtering probes with SNPs | 96,756 |
| Filtering probes that align to multiple locations | 11 |
| Filtering probes located on X, Y chromosome | 16,674 |

SNP, single nucleotide polymorphism.

Table S2. The demographic characteristics of the participants in the discovery cohort.

| Characteristics | AS  (n=30) | HC  (n=15) | p |
| --- | --- | --- | --- |
| Male, n (%) | 27 (90.0) | 12 (80.0) | 0.384 |
| Age, year, mean ± SD | 34.53 ± 9.92 | 32.13 ± 9.42 | 0.441 |
| HLA-B27 positive, n (%) | 23 (76.7) | 6 (40.0) | 0.015 |
| Smoking, n (%) | 11 (36.7) | 6 (40.0) | 0.828 |
| Duration, month, mean ± SD | 94.28 ± 48.79 | - | - |
| NSAIDs, n (%) | 23 (76.7) | - | - |
| DMARDs, n (%) | 5 (26.7) | - | - |
| TNF inhibitors, n (%) | 13 (43.3) | - | - |
| BASDAI, mean ± SD | 6.85 ± 2.03 | - | - |
| BASFI, mean ± SD | 4.46 ± 2.22 | - | - |
| ASDAS-CRP, mean ± SD | 4.36 ± 0.79 | - | - |
| ESR, mm/H, median (IQR) | 29.5 (12.5-46.5) | - | - |
| CRP, mg/L, median (IQR) | 21.1 (12.4-44.0) | - | - |

* P-values were calculated with HCs as references. AS: ankylosing spondylitis; HC: health control; SD: standard deviation; Smoking includes current and past smoking status; NSAIDs: nonsteroidal anti-inflammatory drugs; DMARDs: disease-modifying antirheumatic drugs; BASDAI: Bath Ankylosing Spondylitis Activity Index; BASFI: Bath Ankylosing Spondylitis Functional Index; ASDAS: Ankylosing Spondylitis Disease Activity Score; CRP: C-reactive protein; ESR: erythrocyte sedimentation rate; IQR: interquartile range.

Table S3. The demographic characteristics of the participants in the validation cohort.

| Characteristics | AS  (n=12) | HC  (n=12) | p |
| --- | --- | --- | --- |
| Male, n (%) | 10 (83.3) | 10 (83.3) | 1.000 |
| Age, year, mean ± SD | 29.92 ± 11.84 | 31.17 ± 5.62 | 0.744 |
| HLA-B27 positive, n (%) | 10 (83.3) | 2 (16.7) | 0.001 |
| Smoking, n (%) | 5 (41.7) | 4(33.3) | 1.000 |
| Duration, month, mean ± SD | 58.29 ± 40.40 | - | - |
| NSAIDs, n (%) | 9 (75.0) | - | - |
| DMARDs, n (%) | 5 (41.7) | - | - |
| TNF inhibitors, n (%) | 5 (41.7) | - | - |
| BASDAI, mean ± SD | 3.37± 2.39 | - | - |
| BASFI, mean ± SD | 2.19 ± 1.93 | - | - |
| ASDAS-CRP, mean ± SD | 2.54 ± 1.17 | - | - |
| ESR, mm/H, median (IQR) | 21 (12.0-45.0) | - | - |
| CRP, mg/L, median (IQR) | 10.3 (1.3-32.0) | - | - |

* P-values were calculated with HCs as references. AS: ankylosing spondylitis; HC: health control; SD: standard deviation; Smoking includes current and past smoking status; NSAIDs: nonsteroidal anti-inflammatory drugs; DMARDs: disease-modifying antirheumatic drugs; BASDAI: Bath Ankylosing Spondylitis Activity Index; BASFI: Bath Ankylosing Spondylitis Functional Index; ASDAS: Ankylosing Spondylitis Disease Activity Score; CRP: C-reactive protein; ESR: erythrocyte sedimentation rate; IQR: interquartile range.
