# Supplementary material for: Integrative blood-derived epigenetic and transcriptomic analysis reveals the potential regulatory role of DNA methylation in ankylosing spondylitis
Source: Arthritis Res Ther. 2022 Jan 5;24:15. doi: 10.1186/s13075-021-02697-3 (PMC8728943; doi:10.1186/s13075-021-02697-3)
Supplement: Supplementary file 2 — Additional file 2: Figure S1. The singular value decomposition (SVD) plots showing the correlation between covariates and primary components (PC). Figure S2. Box plots showing the methylation level of differentially methylated CpGs (DMPs) in the discovery cohort. Figure S3. Characteristics of the differential DNA methylation positions (DMPs) associated with AS in the validation cohort. Figure S4. Venn diagram depicting the overlaps between the discovery and validation cohorts. Figure S5. Box plots showing the methylation level of differentially methylated CpGs (DMPs) in the validation cohort. [file 13075_2021_2697_MOESM2_ESM.docx]

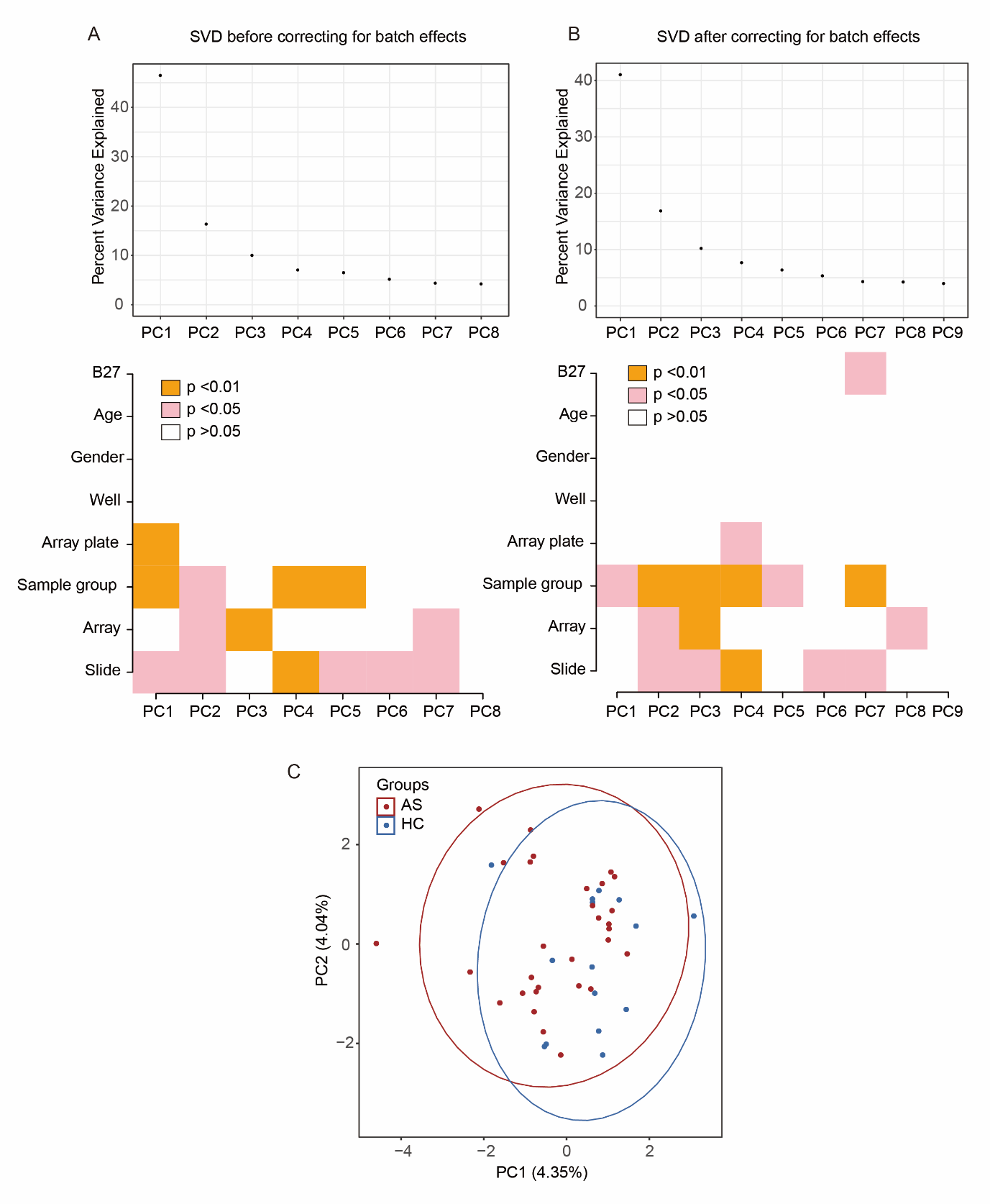


**Figure S1** The singular value decomposition (SVD) plots showing the correlation between covariates and primary components (PC). **A** and **B** show the SVD before and after correcting for the batch effects. The covariates include technical and biological factors. The significance is calculated with Kruskal-Wallis test for categorical covariates and linear regression for numeric covariates. **C**, Principal component analysis (PCA) of top 1000 most variable CpG sites of AS cases form health controls (HCs).


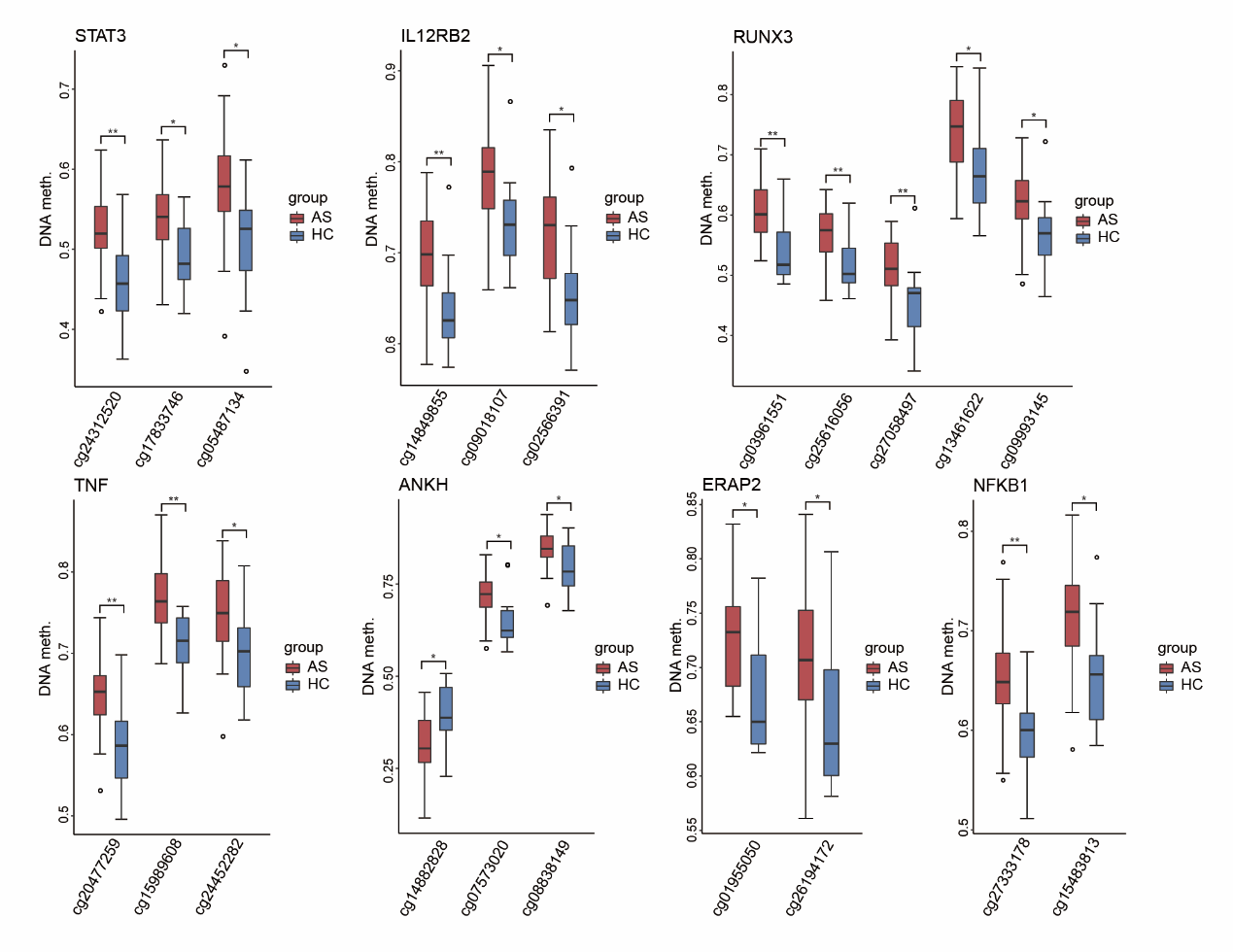


**Figure S2** Box plots showing the methylation level of differentially methylated CpGs (DMPs) in the discovery cohort. These DMPs located genes were confirmed to involve in the development of AS. *P < 0.05; **P < 0.01; ***P < 0.001.


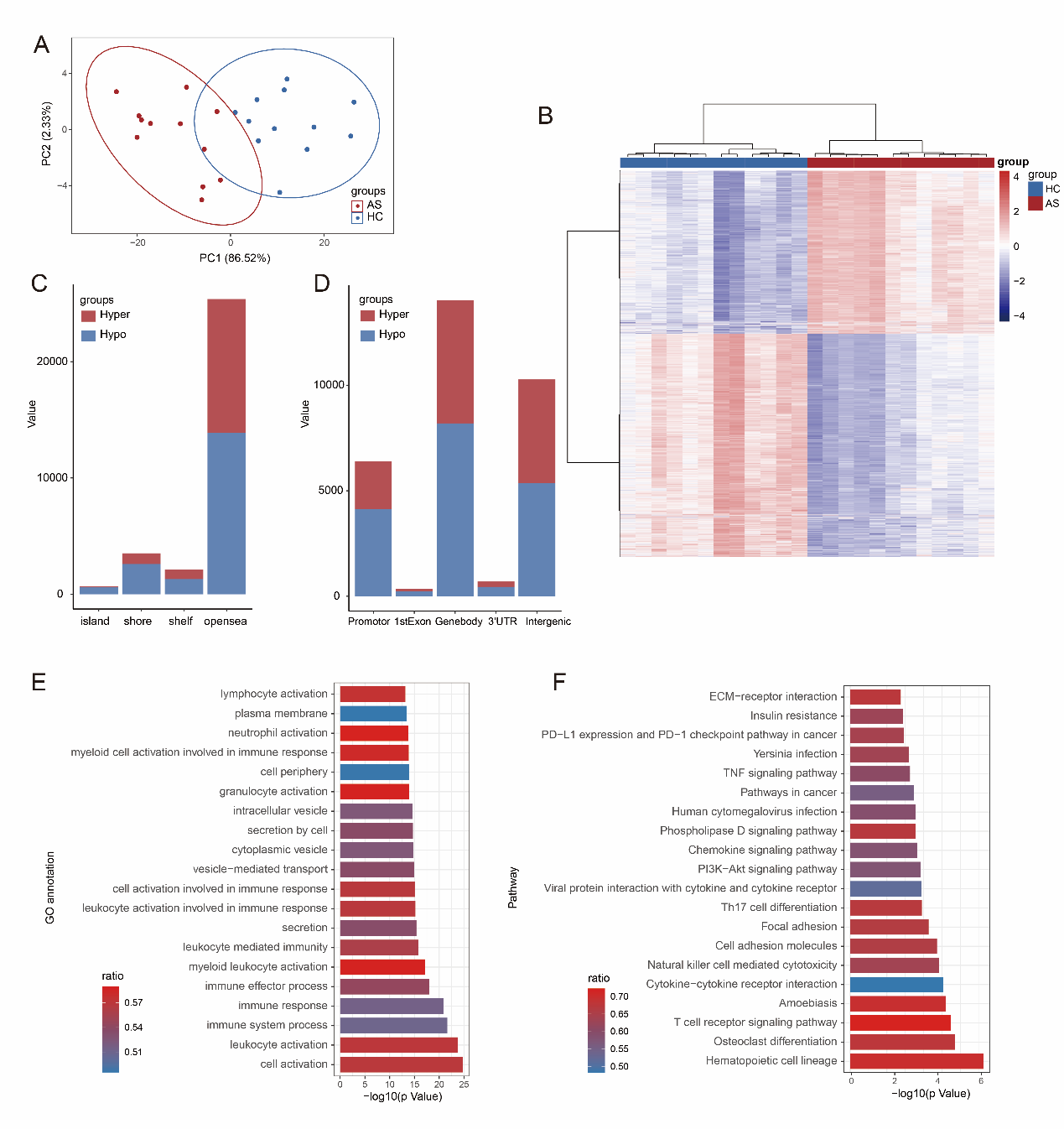


**Figure S3** Characteristics of the differential DNA methylation positions (DMPs) associated with AS in the validation cohort. **A**, Principal component analysis (PCA) of DMPs show separation of AS cases form health controls (HCs). **B**, DNA methylation heatmap showing differentially methylated CpGs between AS patients and HCs. **C**, The distribution of hypermethylated and hypomethylated DMPs relative to CpG island regions. Shores are defined as the 2 kb away from CpG island and shelves as the 2 kb outside of a shore. Regions outside this 4 kb stretch are referred to as the ‘open sea’. **D**, Genomic location of the hypermethylated and hypomethylated DMPs relative to promoters, 1^st^ Exon, gene body, 3’UTR and intergenic. **E**, Representation of the top 20 Gene ontology (GO) enrichment analysis biological process terms of DMPs. Ratio represents the percentage of DMPs in each term or pathway. **F**, Representation of the top 20 KEGG biological pathways associated with DMPs.


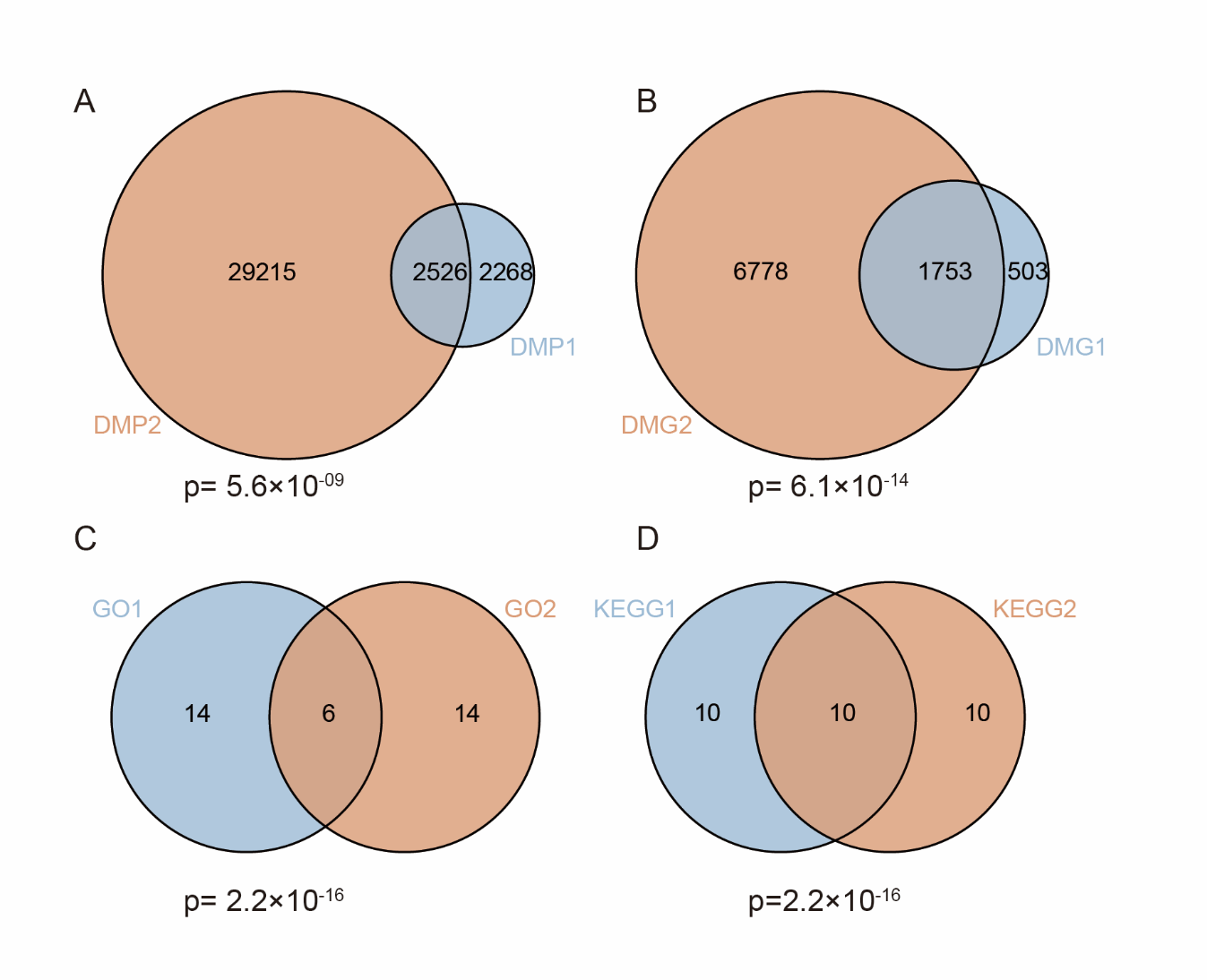


**Figure S4** Venn diagram depicting the overlaps between the discovery and validation cohorts. **A**, differentially methylated probes (DMPs). **B**, differentially methylated genes (DMGs). **C**, top 20 Gene ontology (GO) biological process terms. **D**, top 20 KEGG biological pathways. Fisher's Exact test was used for the significance of overlaps.


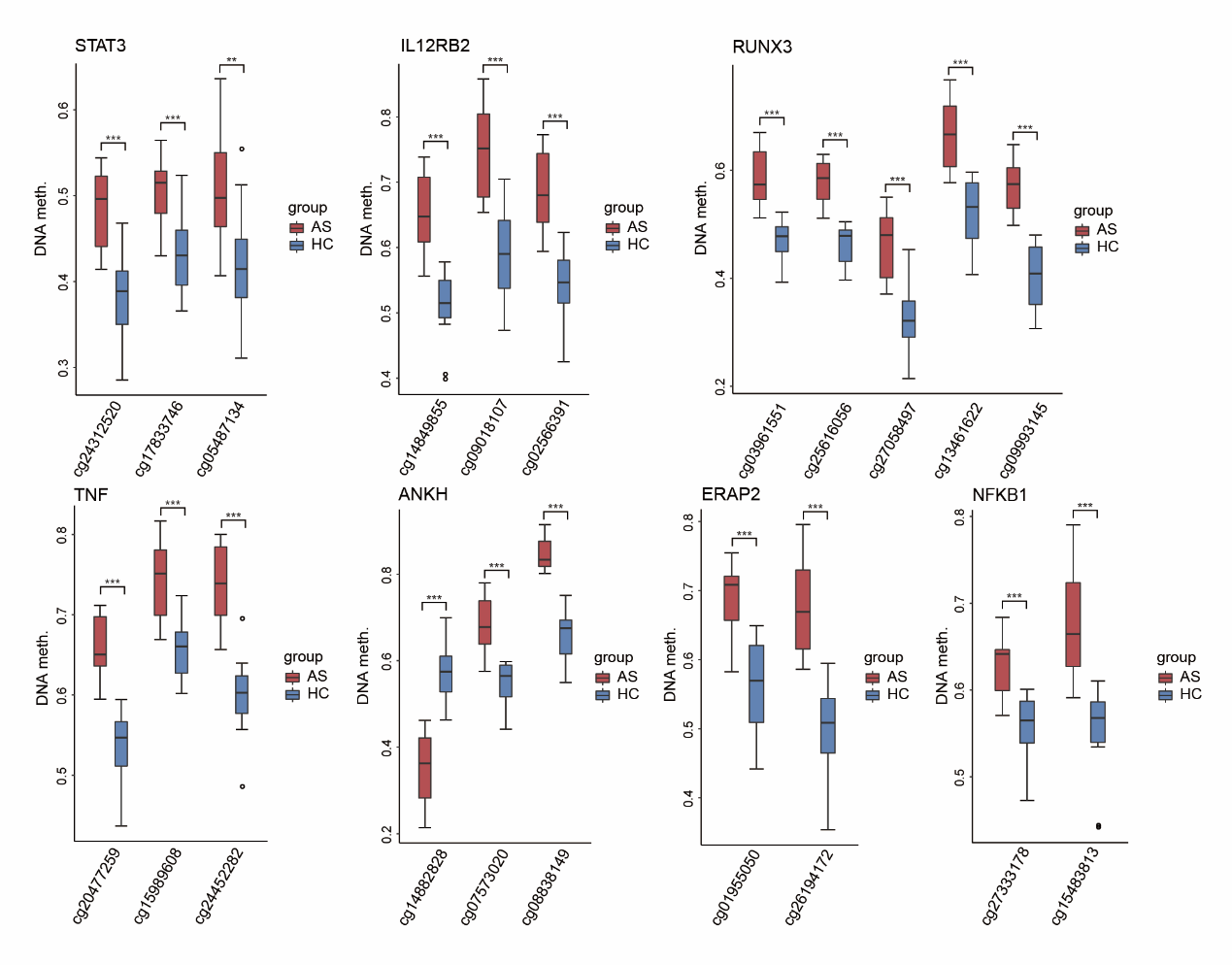


**Figure S5** Box plots showing the methylation level of differentially methylated CpGs (DMPs) in the validation cohort. These DMPs located genes were confirmed to involve in the development of AS. *P < 0.05; **P < 0.01; ***P < 0.001.
